# The Romani Minority, Coercive Sterilization, and Languages of Denial in the Czech Lands

**DOI:** 10.1093/hwj/dbx033

**Published:** 2017-08-17

**Authors:** Sarah Marks

**Affiliations:** Birkbeck, University of London

## Abstract

Sterilizations of Romani women in socialist Czechoslovakia, either carried out without proper consent, or coerced through substantial financial incentive, were first reported in 1978. Yet these practices did not end with the fall of communism, and it took until 2005 for this to be officially acknowledged by the Czech government. This article draws on published and unpublished documents, as well as oral history interviews, to trace the history of efforts to expose such practices, ‘come to terms’ with their existence, and change social attitudes in relation to the Romani minority in the Czech lands. These exposures have uncovered instances of denial, and have also offered up a variety of ways of understanding the mental and social mechanisms that might have enabled silences, refusals or disavowals with regard to human rights abuses. Under Communism, dissidents associated with Charter 77 elaborated these through the philosophical concepts of phenomenology; after the transition to democracy, a more psychological and therapeutic language came to the fore. I argue that the Czech case suggests that the historiography of denial and disavowal could be enriched by looking beyond the framework of psychoanalysis: by taking into account how historical actors, sometimes with opposing worldviews, have comprehended these processes within the languages of their own culture and period.

## ***

Sterilizations of Romani women in socialist Czechoslovakia, either carried out without proper consent, or coerced through substantial financial incentive, were first reported in 1978. Yet it took until 2005 – twenty-seven years later, and long after the fall of communism – for this to be officially acknowledged.^1^ In his report from that year, the Czech Republic’s ombudsman admitted that the practices had by no means come to an end with the transition to democracy, and complaints were still being filed. It took a further four years for the government to make an official statement of apology.^2^ In 2015, Czech ministers rejected a bill that proposed the provision of compensation to women who had been illegally sterilized, along the lines of parallel initiatives in Sweden and Switzerland. They argued that doctors and social workers had taken the initiative, and so the state was not responsible.^3^

Awareness of these coercive sterilizations – and indeed the wider situation of Roma in the former Czechoslovakia – remains limited. When these matters have been brought to light, whether by academic researchers, human-rights organizations, or prominent figures within law and politics, the impact has remained marginal.^4^ But the commentaries, however few, have uncovered instances of denial. Some have also offered up a variety of ways of comprehending the mental and social mechanisms that might have enabled silences and refusals in regard to the rights of the Roma. This article draws on published and unpublished documents, as well as oral history interviews, to trace the history of efforts to expose such practices, ‘come to terms’ with their existence, and change social attitudes in relation to the Romani minority.^5^ The debates around the coercive sterilizations, and the ways in which they were exposed, serve as a prism through which to examine how processes of denial and recognition have been described in Czech culture. These languages were, in turn, mobilized by campaigners and academic commentators as a means of protest, and to promote an ethic of inclusive civic responsibility.

Three stories are central to what follows. All concern whistleblowing attempts during the Communist period and after. The first endeavour culminated in the publication of a key document in 1978 by the dissident organization Charter 77. This reflected the philosophical framework of Czech phenomenology, drawn from European thinkers such as Husserl, Masaryk, Heidegger and Arendt, which formed the ethical underpinning for their programme of action. These dissidents argued for the ‘uncovering’ and ‘bringing into awareness’ of breaches in the protection of human rights, and made use of a language built around challenging disavowal and concealment, both at the level of high politics and in the lives of everyday citizens. This way of understanding efforts to deny human-rights abuses, and the implicit failures to recognize or speak out about them, fell outside of the familiar psychoanalytic discourses that have been noted, and indeed appropriated, by authors writing on the historiography of denial.^6^ This raises questions about how historical actors from non-Freudian intellectual and cultural traditions have framed problems of seeing and not-seeing, or avoidance of knowledge about violence and the violation of human dignity. I argue that this language of phenomenology offers an example of how our understanding of denial as a social and mental process could be enriched by looking beyond psychoanalysis as the central explanatory framework, by paying attention to alternative accounts developed in different historical contexts.

The second attempt to expose the sterilizations, begun in 1988, resulted in a report drawn up during the collapse of the Communist regime, with financial support from the Dutch government. Although it exposed widespread abuses, it failed to motivate change. The absence of action that followed this alarming exposé was all the more striking given that it was published shortly after the Velvet Revolution in 1989. The findings of this report were overlooked and minimized, even though a number of prominent figures from the Charter 77 movement, who had made human rights the core of their political programme, had gained ministerial positions.

Finally, when the Public Defender of Rights officially investigated and confirmed reports of coercive sterilizations in 2005, a new rhetoric emerged – one which used psychologically inflected concepts of denial, trauma and collective memory. I argue that this was illustrative of the increased stake that the psy-disciplines had gained in Czech society by the twenty-first century. Along with other, contemporaneous literature by Czech authors on the Romani minority, this shows how therapeutic knowledge came to play a role in post-socialist life, beyond the realm of the individual. Politicians and activists appropriated it in pursuit of the goal of a *collective* psychological transformation, and latterly, to call for a more inclusive democratic national community.

## THE ROMA IN CZECHOSLOVAKIA

In the early 1970s, the Romani population was estimated to be around 2.5 per cent of the total Czechoslovak population, with numbers approaching 300,000.^7^ The majority resided in the Slovak parts of the nation, as much of the population in Bohemia and Moravia perished during the Nazi occupation. During the war, killings were most often organized as round-ups and shootings in the Czech towns, but many also died in concentration camps at Auschwitz, or the camp at Lety, south-west of Prague, built specifically to incarcerate Romani people.^8^

The state’s stance towards the minority for much of the Communist period was orientated around a refusal of their cultural identity.^9^ In 1958, decrees were issued which limited nomadic movement and actively committed Czechoslovakia to a policy of assimilating the Roma, in part by restricting travel and establishing settlements. Although there was a short period of official recognition of the Roma as an ethnic group after the Prague Spring in 1968, by the mid 1970s, as I discuss below, the state had essentially begun to disavow their existence, shutting down organizations which represented their interests and preventing academic research into Romani culture.^10^

The first known instances of coercive sterilization of women from the Romani community occurred in 1970s, with numbers increasing in subsequent decades.^11^ At no point was there a clear, nationally-endorsed policy prescription to enforce these practices, directed specifically at the Roma. But the reports discussed in this article have indicated that local authorities were ethically implicated, by a culture which incentivized social workers to coerce Romani women into giving consent. This was in addition to an established medical literature that advocated eugenic thinking with regard to the control of the Romani population.^12^ Conversely, sterilization was a method of family planning that was barely used by ethnically Czech and Slovak women, and health authorities did not promote this procedure as an option to the majority population.^13^

The late 1970s, when our story starts, is characterized as the ‘normalization’ period in Czechoslovak history. After the reformist zeal of the Prague Spring, the Soviet Union invaded in August 1968 to restore order and, in their words, a state of normality. This went along with an increase in the use of the security services to quell opposition and monitor swathes of the republic’s citizens. The state attempted to go some way to compensate for the restrictions on freedom by improving access to consumer goods. This was also the period – in Czechoslovakia as well as elsewhere in the Soviet sphere – when political dissent became more conspicuous at home and abroad. In the Czechoslovak case, the most recognizable dissident movement came into being in January 1977, with the publication of Charter 77 by a group of Prague writers and campaigners in protest against the state’s worsening record with regard to human-rights abuses.^14^

## CHARTER 77 DOCUMENT 23: HOLDING UP A CRITICAL MIRROR

On 14 December 1978, signatories of Charter 77 circulated its twenty-third campaign document, ‘On the position of Romani fellow citizens submitted as a basis for public discussion’.^15^ The human-rights campaigner Jan Ruml assembled the evidence for this report.^16^ The document itself was drafted by Zdeněk Pinc, a philosopher in the phenomenological tradition, and one of many academics forced out of university positions by the state in the 1970s.^17^ The Charter’s spokesmen, the playwright Václav Havel and the philosopher Ladislav Hejdánek, provided a foreword effectively endorsing its contents.

From its very first sentence, Document 23 called out the dangerous lack of acknowledgment given to the Roma – the ‘most discriminated against minority’ – in public discourse. ‘That ignorance [literally ‘uninformedness’, from *neinformovanost*] is the consequence of purposeful concealment of everything substantial relating to the Roma. The severity of the situation has gone so far that it cannot continue without protest.’^18^ This state-fostered uninformedness gave rise to racism, segregation and further disenfranchisement. Pinc observed a process akin to dehumanization in the ‘public imagination’: the Roma were never judged to be victims of injustice – that was a privilege reserved for those considered to be ‘decent people’.^19^

Looking back to the earlier history of the twentieth century, Pinc warned of the consequences of this mental representation of minority groups within the collective imagination of the majority:

If silence about these matters continues, it could lead to a tragic paradox: Gypsies-Roma will merge in common awareness – in the awareness of civically apathetic consumers just as much as in the awareness of citizens advocating legal justice – with social villains… And the old Jewish role will be reprised with a new cast, a reprisal which has in fact already started.^20^

The state’s own policies appeared to contribute to this reprise: by refusing the Roma the status of a minority nation, and instead ascribing to them the designation of ‘ethnic group’ (*etnická skupina*), the government did not afford them equal rights to other constituent groups within Czechoslovakia. After all, Czechs and Slovaks were legally categorized as autonomous ethnic ‘nations’ whose language and culture deserved protection. This was an outright rejection of the very existence of the Roma on their own terms.

The dissidents claimed that a recent change of policy had hardened attitudes. After the Prague Spring of 1968, the authorities had actually taken a step towards formally acknowledging the existence of the Roma as a group (albeit not as a nation), by allowing for the establishment of an ‘Association of Gypsies-Roma’ within a state framework of civic organizations. At that time, Czechoslovakia was one of only three Communist states in Europe (along with the USSR and Yugoslavia) to bring ethnic groups into state structures. But this was completely reversed in 1975, when the Association was dissolved. There followed, as the political scientist Peter Vermeersch puts it, a ‘shift towards negation and assimilation’, underscored by official rejection of attempts to gain recognition of nation status for the Roma.^21^

This rapid about-turn appeared all the more egregious given Czechoslovakia’s pledge to honour human rights in the internationally agreed Helsinki Accords of the same year.^22^ A key moment for the brief Cold War détente between the American and Soviet blocs, two of the ten articles in the Accords committed participating countries to guarantee ‘respect for human rights and fundamental freedoms, including the freedom of thought, conscience, religion or belief’ and ‘equal rights and self-determination of peoples’.^23^ Document 23 pointed out that this rendered ‘the factual and legal situation of the Roma… full of contradictions’.^24^ Despite all the state’s pledges to adhere to covenants on the world stage, ‘the rights of the Roma as a minority… are de facto denied’.^25^

Pinc employed the phrase ‘juristic alibism’ (*juristická alibismus*) to describe the authorities’ ambivalent actions during the 1970s. In Czech, this phrase implies an effort to avoid responsibility through blame-shifting, or by behaving in a ‘two-faced’ or duplicitous manner. He suggested the state had failed to take responsibility for ameliorating conditions and instead shifted the blame onto the Roma. In turn, while there were some policies to improve housing, these were in effect cancelled out by ‘obviously…unconstitutional’ measures in some regions to restrict the Romani people’s freedom of movement and choice of employment.^26^

One of the starkest inconsistencies was apparent in practices of population control. The state performed sterilization as though it were a ‘planned administrative practice’, with social workers’ performance evaluated by how many women they had managed to get to consent to the procedure.^27^ This ‘consent’ was, however, ‘obtained through influence, frequently through demagogic and exploitative cash rewards, and its objectivity not guaranteed’.^28^ The Chartists saw this as clear evidence that sterilization was being used as a measure of control in the interests of ethnic Czechs and Slovaks, directed against the very existence of the Romani minority. If Czechoslovak institutions did not face up to such matters, the dissidents argued, they would find themselves contravening the state’s own Criminal Code. Article 259 concerned The Law Regarding Genocide:

1. Whosoever intentionally destroys, wholly or in part, any national, ethnic, racial or religious group by carrying out measures intended to prevent births in such a group… can be sentenced to 12–15 years in prison, or the death sentence.^29^

The scholar of human-rights law Helen O’Nions argued retrospectively that it would have been difficult to prove that the sterilizations were an act of genocide. In terms of the ‘intention to destroy’, the authorities could disclaim such an allegation, as they simultaneously pursued policies to improve welfare and housing for the Roma elsewhere.^30^ The dissidents’ accusation of ‘alibism’ appeared to hold true.

For the Chartists, the state’s denial of the rights and freedoms of the Roma as a nation, and the practice of coercive sterilization, offered a salient case in point for their larger remonstrations against the regime. They could use the anomalous and prejudicial treatment of the Roma as part of a more all-encompassing critique of the state and its failures to abide by internationally agreed commitments, as well as, in some cases, doubtful adherence to its own national legal framework. The ostentatiously public declaration of agreement with the Helsinki Accords was an incongruently grand gesture, by contrast with the frequent, ubiquitous failure to uphold basic human rights on a day-to-day basis in Czechoslovakia. Zdeněk Pinc’s framing of the debate in terms of mechanisms of ‘concealment’, ‘uninformedness’, ‘merging in common awareness’ and ‘alibism’ coheres with the Chartists’ broader philosophical programme, which was shot through with concepts of denial and unseeing.

The Party functionaries themselves, as far as the Chartists were concerned, were in some ways cynically aware of their hypocrisy, and motivated primarily by the perpetuation of their own power. Ordinary citizens, on the other hand, were manipulated into acceding to this farcical state of affairs at significant personal cost. In order to live under such a regime, some sort of mental process of concealment, of self-deception, became necessary. And by engaging in this, they became both the ‘victims’ and the ‘pillars’ of the lie.^31^ The circulation of the Charter itself in January 1977 was a call for ordinary men and women to step out of their automatic habits, to face up to their collusion with the state’s dishonesty, and take responsibility by calling the authorities out. In the words of Jan Patočka, the philosopher and one of the founders of the original Charter, ‘it is not pleasant to be jarred into awareness, out of our comforting illusions’.^32^ But the only thing that could lead to a destabilization of the regime’s control, he argued, was a collapse of their confidence, ‘a realization that their acts and injustice and discrimination do not pass unnoticed, that the waters do not close over the stones they throw’.^33^

## THE PHENOMENOLOGY OF DENIAL

Document 23 provided an account of Czechoslovakia’s disavowal of its mistreatment of the Roma that was rooted not in a psychoanalytical model of the mind, but rather in the specific philosophical tradition of Czech phenomenology. The three men who wrote or signed the document – Pinc, Havel and Hejdánek – all identified with this school. To fully understand the arguments at play in Document 23, we need to place it in its immediate intellectual context. Pinc and Havel were both students of Jan Patočka, who had died suddenly in March 1978 following a prolonged police interrogation.^34^ Their texts drew from an eclectic range of Ancient Greek and existentialist philosophy, and particularly Heidegger’s *Being and Time*.^35^ They adopted the label of ‘phenomenologists’ in part to locate themselves within a particular Central European intellectual heritage, which included philosophers such as Franz Brentano and the interwar Czechoslovak president Tomáš Masaryk. They were also paying homage to Patočka’s status as a recognized philosopher in the international phenomenological tradition, as he had studied with Edmund Husserl and Martin Heidegger.^36^

For the Czech phenomenologists, the ability of all persons to differentiate between good and evil was an essential quality of humanity. Individuals could nevertheless act in evil ways through a lack of awareness, whether through wilful or unavoidable ignorance, which Patočka termed ‘blind wandering’ (*bloudění*). This was exemplified in the myth of Oedipus who, although a man of ethics, killed his father and married his mother through a lack of knowledge of his own circumstances.^37^ In this vein, Pinc’s Document 23 underlined the necessity of exposing the state’s ‘concealment’ of the precarious situation of the Romani minority to the general populace. Unless they were made aware of the reality, even citizens of good conscience might repeat the same prejudices and actions that had made possible the anti-Semitic violence of earlier decades, only this time directing it towards the Roma.

There were other mechanisms by which an individual might come to ‘blindly wander’. Czech phenomenologists drew on Heidegger and Hannah Arendt in their use of the concept of ‘authenticity’, from which the dissident phrase ‘living in truth’ was adapted.^38^ Echoing both Heidegger and Husserl, they cautioned against the privileging of technology and scientific rationality in modern societies, which limited the human horizon by revealing only the facet of truth that could be described by objective science. This offered a denigrated picture of nature and alienated humans from authentic life. As Aviezer Tucker has shown, the Czech dissidents drew inspiration from their Russian counterpart, Alexander Solzhenitsyn, who argued at Harvard in 1978 that modern scientific rationalism overlooked the existence of evil, and in doing so, allowed it to ‘operate unnoticed and unchallenged’.^39^ While Western democracies were every bit as culpable in this regard, the ‘scientific socialist’ systems of Eastern Europe were peculiarly adept at creating impersonal bureaucracies, in which individuals might reasonably be drawn to the ‘strange hypnotic allure’ of ideology as a means of survival.^40^

This critique became a leitmotif of the Charter movement. It was threaded through various samizdat tracts, most famously in Havel’s essay *The Power of the Powerless*.^41^ Havel personified the figure of the citizen who shared the blame for the moral degradation of society in his portrait of a greengrocer, who perennially displayed a sign in his window reading ‘Workers of the World, Unite!’. The sign would be delivered to him regularly with his fruit and vegetables and he would uncomplainingly display it. He justified it to himself thus: why not display such a sign, if it keeps one out of trouble – a real and ubiquitous risk under the Communist regime – especially when it is basically an inoffensive message? In Havel’s words, ‘the sign helps the greengrocer to conceal from himself the low foundations of his obedience, at the same time concealing the low foundations of power … behind the facade of something high’. This was ideology, the rituals and sloganeering ubiquitous to everyday life under socialism. These provided ‘the illusion of an identity, of dignity, and of morality’ which ‘enables people to deceive their conscience and conceal their true position and their inglorious *modus vivendi*, both from the world and from themselves’. The process by which the greengrocer made sense of his act was, for Havel, a ‘psychological excuse’ that enabled power to constitute itself ‘inwardly’. His language brimmed with metaphors of seeing and unseeing: the individual ‘hides’ his fallenness from himself behind a ‘veil’ of ideology. Political phrases ‘cloak’ the true intentions of power-driven Party members.^42^ These were the same dynamics that exacerbated the situation of the Roma. Ordinary citizens did not intervene, not only because of an ‘uninformedness’ in part encouraged by the state, but also to some extent through a voluntary turning away. Document 23 was one of many Charter testaments that sought to make people face a reality of injustice that they would rather push out of their awareness, and to spur them on to address it.

At no point do texts by Havel – nor indeed by Pinc or Patočka – invoke the concept of the unconscious, although there are parallels with Freudian ideas of denial and disavowal. Nor is there evidence to suggest, for instance, the direct import of contemporaneous ideas derived from Theodor Adorno and Max Horkheimer, or from the emerging German literature on ‘coming to terms with the past’ (*Vergangenheitsbewältigung*) which promoted a similar ethic, albeit more explicitly psychoanalytically oriented.^43^ In fact, Patočka actively rejected Freud’s conception of the unconscious because of its suggestion that there was discontinuity within the psyche. He favoured an understanding of the human mind as not divisible into enduring structures such as the id, ego and supergo; consciousness was more fluid, and subject to temporal and historical flux.^44^ But even without recourse to Freudian defence mechanisms such as repression, rationalization or splitting, this framework for understanding denial did emerge in Czechoslovakia in the 1970s – and it was shaped as much by the intolerable pressure of political circumstances as by engagement with European philosophical traditions.

## THE SURRENDER OF FERTILITY

The Czechoslovak state never officially responded to Document 23. The matter of coercive sterilization was not publicly addressed and the practice continued to proliferate. But although in its home country the document languished mostly in obscurity, it reached a limited readership abroad, via a Charter 77 support group in the Netherlands, which enabled a further campaign to come to light a full decade later.

In spring 1988 Paul Öfner, a Dutch journalist, was visiting the Czech countryside as a tourist. Following his training in social anthropology, Öfner had worked with gypsy communities within the Netherlands, and was interested to find out about the situation of the Roma in Czechoslovakia.^45^ He contacted Milena Hübschmannová (1933–2005), a linguist who had earlier begun to establish the field of Roma Studies at Charles University in Prague. This was interrupted after she was forced out of her position in the pedagogical faculty for opposing the state’s policy of assimilation.^46^ From the 1950s on, Hübschmannová had travelled extensively across Czechoslovakia to compile folktales and songs. She had begun a systematic study of Romani dialects, motivated by her fear that the official necessity for using Czech and Slovak would result in the eventual disappearance of these languages altogether.

By the time Öfner made contact in the late 1980s, Hübschmannová was increasingly marginalized within academia, and she remained so until the fall of Communism. She found employment at a Prague language school in the meantime. Her home, however, still provided a hub where students of Romani culture and language congregated. News of the sterilization practices had reached the group, and they welcomed Öfner. He recalls, ‘it was she who convinced me, because I asked if it was helpful, or perhaps even dangerous, to get involved in this question. And she said that, no, it was the only way out, because they [the state] tried to hide it and, “we are not in a position to come out into the open with the things we know”. She had already had trouble with the regime’.^47^

Despite the risks, two students of the Romani language, Ruben Pellar and Zbyněk Andrš, had previously started to investigate the rumours by travelling to affected villages in the eastern regions of Czechoslovakia. With the assistance of two female colleagues, Edita Žlnayová and Hana Šebková, they began structured interviews with women who had undergone operations, and uncovered evidence of monetary rewards being used to incentivize women to undergo sterilization. These offers could reach as much as 25,000 crowns: to put this in perspective, Pellar’s own salary as an information retrieval worker in a Prague medical library was 2,000 crowns per month. Both wanted to conduct further research to uncover the extent of the problem, and to end the practice. In Pellar’s words, ‘I was outraged. I asked myself, how is it a possibility, in a state where the population is decreasing, that the state can pay people for not having children?’.^48^

Paul Öfner returned to the Netherlands and, along with his colleague Bert de Rooij, sought out funding to support Pellar and Andrš in expanding their research. This was facilitated by Jef Helmer, then president of the Dutch-based ‘Information on Charta 77’ foundation, which supported the interests of the Czechoslovak Charter signatories. He was also involved with the Association Lau Mazirel which campaigned for the rights of Roma gypsies and other nomadic peoples.^49^ Helmer was already aware of the issue of coercive sterilization thanks to Document 23. In 1987, ‘Information on Charta 77’ were contacted by activists in Sweden and the United States, who asked whether there were any updates about these practices since the first exposé. Unable to answer the inquiries these correspondents made as to the extent of the problem, Helmer went about ‘getting some money for more-or-less secret research. The Dutch government, quite openly… agreed to finance this mission’.^50^

*The Surrender of Fertility* (*Het Afkopen van Vruchtbaarheid*) was published in Amsterdam in June 1990.^51^ Drawing on interviews with 123 women between 1988 and early 1990, the report found many had not been properly informed about the irreversibility of the procedure or how it was performed, nor were they given information about alternative, ‘less intrusive’ forms of contraception. The authors also alleged that there were some cases where representatives of the regional governments (*Národní výbor*) refused women other financial benefits, such as child support, assistance and maternity benefits, or the signing of building permits, if they refused to give consent to sterilization. The report described how ‘rent debt’ was used to motivate consent, and one case in which a twenty-two-year-old woman was threatened with the removal of her only child if she did not undergo the procedure. Cash incentives were offered by employees (usually social workers) of the regional government’s ‘Gypsy Committees’, sometimes worth up to nine months of the average salary, although women sometimes reported that they received substantially less money than had been promised.^52^

The report pointed out a startling incongruity in family planning policy: sterilization procedures were not recommended to the Czech and Slovak population.^53^ This was a measure directed specifically at the Romani community. It also documented justifications for the regulation of the reproduction of the Roma in East Slovakia given by medical doctors in a 1989 issue of the journal *Zdravotnická pracovnice* (*Health Worker*, a journal published by the Czechoslovak Medical Society). This article claimed that, ‘these are citizens who evince a mostly negative attitude with regard to work and education, a high crime rate, a tendency to alcoholism, and their wives to promiscuity… and who, for the most part, lag behind the social and cultural developments of other groups of the population’.^54^ The report thus concluded that these practices could not be explained as a consequence of the communist system, but were rather a manifestation of widely-held cultural attitudes within Czechoslovakia.^55^

## THE RECEPTION OF THE 1990 REPORT

The story was taken up in the Dutch media. Öfner secured the cover feature of the daily newspaper *Trouw*, as well as detailed articles in the Dutch Roma magazine *o Drom.*^56^ Öfner suggests that one reason for the interest, and shock, expressed in the Netherlands was that it resonated with collective memory of forced sterilizations conducted by the Nazis there during the Second World War.^57^ By stark contrast, the campaigners faced significant difficulty in raising interest within Czechoslovakia itself. This was all the more shocking given that the report was published after the fall of the communist regime, at a time when freedom of speech became protected, and changes in state policy were now possible. Even though it had become easier to ‘act publicly’ and conduct research legally, in Pellar’s words:

We asked many organizations to help us: the Red Cross, women’s organizations, the Communist Party. There was, in effect, no answer, except for one. There was a state-supported Committee for Human Rights, and one man reacted positively to our letter.^58^

This letter initiated an investigation on the part of the Czechoslovak Prosecutor, published in 1990. It found that coercive sterilizations, sometimes without any consent, had indeed occurred in locations across Bohemia and Moravia. The authorities had, allegedly, attempted previously to claim it was confined to the area around Ostrava. The letter concluded that local medical authorities should monitor what had happened and assess the legality of sterilizations, and proposed that the Chief Expert for Gynaecology and Obstetrics (a governmental advisory office) should draft changes to the law on sterilization. There is no evidence that either recommendation was followed through.^59^ Campaigners found it difficult to engage the medical community or broader civic or political organizations on the issue. In 1988, Ruben Pellar wrote to the main Czech medical journal, *Časopis lékařů českých*, to say that the practice of giving money for sterilization should be stopped, and provided evidence of coercion. The journal editors rejected his letter for publication because it was ‘non-scientific’. Pellar reflects that, ‘when we tried to raise awareness there was often no reaction. I believed, perhaps naively, that if we exposed the practice, it would lead to its end. Instead, you had the feeling you were speaking to an empty room’.^60^

Jef Helmer, by then a lecturer in social work at a Prague higher education college, had begun to work with Czech and Slovak colleagues to reinstate social work training in Prague, Brno and Bratislava after 1989. He had experienced similar difficulties in galvanizing people to investigate or protest on the issue.^61^ He continued to be engaged with the Charter 77 signatories, some of whom had come to hold ministerial office or to have significant political roles in the newly democratic republic:

I remember that in May 1990 there was a big conference in the Netherlands, and there were delegates from Eastern European countries with… prominent former dissidents. I confronted them with this report and they said they didn’t know if it is all true, or what kind of research methods they used…so they tried to make it relative, to play it down. I was surprised… Some weeks later I was in Prague and I spoke to a high official in the Ministry of Social Affairs. Now he didn’t deny the report, but said it was maybe partly true, or maybe ten per cent true. So he also tried to play it down.^62^

Beyond the Netherlands, there was also a lack of international interest in holding the Czechoslovak government to account on this. Within Czechoslovakia, the post-communist period saw a rise in ethno-linguistic nationalism. This ultimately precipitated its separation into independent republics and facilitated the rise of political parties with anti-Roma platforms. Changes in policy also resulted in a removal of the state’s obligation to provide work to all citizens. Roma communities faced prejudice and discrimination from potential employers, worsening their social and economic marginalization.^63^ The lack of attention paid to the problem of coercive sterilization should be seen within this frame: while democratic institutions and free market economics were beginning to take root in the newly independent nations, these rapid transformations did little to open up space for the recognition of Roma rights in the immediate term.^64^

## RECOGNITION IN THE POST-COMMUNIST PERIOD

As with Charter 77 Document 23, *The Surrender of Fertility* report received little official acknowledgement, despite the fact that the post-transition establishment’s political heritage was grounded in the defence of human rights. Many former dissidents had now come to assume political office. The investigation did, nonetheless, provide a foundation for later efforts to bring the Roma sterilizations to light. Although the process was slow, the opening of civil society after the fall of communism did enable a number of Roma activists to campaign publicly. They established and co-operated with both national and international non-governmental organizations.^65^ Alongside the provision of legal assistance for submitting official complaints, this finally resulted in wider recognition of coercive sterilizations in the early twenty-first century. New reports called for an explicit acknowledgement of the practice after the fall of communism. These campaigns also began to articulate the need for a transformation of attitudes both towards the Roma, and individual reproductive freedom as a principle in itself, so as to prevent the conditions which had enabled the sterilizations in the first place.^66^

In 2005, a major investigation was launched by the Czech Defender of Rights, or Ombudsman, Otakar Motejl. He was a non-partisan lawyer who, although not a signatory of the Charter, defended a number of dissidents during the communist period. Motejl called not only for a ‘coming to terms with’ the ‘existence’ of the coercive sterilizations, but also for a proper reckoning with the fact that these actions had been justified by eugenic reasoning.^67^ Drawing on recent research by historian Michal Šimůnek, he also argued that people must ‘confront’ the overlooked fact that a significant eugenics movement had existed from the interwar period ‘even [in] Czech society’. Motejl proposed that the arguments in favour of negative eugenic logic needed to be publicly challenged.^68^ The coercive sterilizations became, in other words, a focal point for wider debates about national ethical and political values. Recognition and cessation of the practice was the primary goal, but also at stake were ideas of nationhood, heritage, and the moral conduct of the community, past and present.

The Motejl report also reflected a growing interest in the psychological mechanisms that might be operating in the way the Czech majority related to the Roma minority. This marked a shift away from the previously predominant focus on human rights, and towards a more diagnostic or even therapeutic approach. For instance, there has been an increasing use of psychoanalytically inspired interpretations of prejudice or denial, and the use of what one may call ‘trauma discourse’. This shift could be understood as a response to – and an attempt to account for – the limitations of human-rights activism to mobilize changes in practice or in attitudes. Despite the exposure of the practices first in 1978 then again in 1990, Motejl emphasized, Czech society had failed even to ‘accept the unpleasant reality’, and for catharsis to be achieved at a national level, it was necessary to fully ‘accept that something intolerable is taking place’.^69^

This growing concern with the psychological dimensions of prejudice and disavowal can be found in the more recent academic and campaign literature on the Roma. In attempting to understand the coercive sterilizations and continued marginalization of the community, external commentators such as Claud Cahn and Nidhi Trehan describe collective processes such as ‘a dynamic of denial’, or the existence of ‘long-term historical patterns, daily consciously or unconsciously re-enacted, whereby the state intervenes as caretaker, effectively demoralizing Roma through paternalism and pressure toward a kind of neutralized conformity’.^70^ Theories of racism now seek to take account of the mental processes of ‘distanciation’ of self and other, or the operation of collective ‘blind spots’.^71^

František Burda, for instance, used the case of the Roma in the Czech lands to consider larger questions about violence in culture through the lens of the French theorist and historian René Girard, as well as the Polish sociologist Zygmunt Bauman. Burda describes the ‘unconscious’ dissemination of negative stereotypes in the media as conforming to a type of exclusion, which, he argues, is itself a psychological defence.^72^ This, it is claimed, is a response to an existential anxiety, the ‘remedy’ for which becomes manifest ‘in identification with one social group and disidentification with the other’. Furthermore, the fact that most Roma are forced, in Burda’s words, to live in enclaves or *holobyt* (a Czech word denoting poor quality, unfurnished social housing) is ‘an external manifestation of symbolical exclusion and a mental ghetto created by the Czech majority society’.^73^

Concern with symbolical exclusion and the operation of mental ghettos has also appeared in historical writing: the Roma holocaust has been seen as an example of a ‘blank space’ (bílá místa) in the historiography of Czechoslovakia. Ctibor Nečas’s work on Romani history builds on the idea of the blank space as a kind of national forgetting, particularly in the official literature under communism.^74^ Historians, then, have taken it upon themselves to write such forgotten truths back in to the collective memory.^75^ The Roma concentration camp at Lety, and the controversy surrounding its memorial, afford important examples for this kind of analysis.^76^ Historians of the wider Central and Eastern European region – particularly with relation to Poland – have begun to examine how questions about the violence of the past have come to be framed in terms of collective memory, trauma, and ‘working through’.^77^ As Stephen Frosh has argued in relation to postwar German attempts to come to term with anti-semitism, this way of approaching the past ‘holds the psychopolitical hope that comprehending it in these terms might offer a route towards the “treatment” or therapy of society’.^78^

This goal is certainly true of the Ombudsman’s report itself, but it has also been espoused most emphatically in the work of Klára Samková, a seasoned Czech politician and human-rights lawyer, and herself a vociferous campaigner for the Romani community.^79^ With the support of senior mental-health professionals her book, *Romská otázka* (the Roma Question), reimagines the issue in terms of existential psychotherapy and collective, intergenerational trauma.^80^ Samková suggests that the behavioural patterns of the Roma community arose as a survival response, invoking Post-Traumatic Stress Disorder as a diagnostic explanation. This, she argues, is a psychological consequence of long-term historical exclusion and prejudice. For Samková, solutions will only come about if an increased role is taken by psychotherapy professionals and a ‘therapeutic’ process is developed involving ‘a complete rebuilding of relationships’ between the majority population and the Roma minority, both of whom suffer through this sense of disconnection. Such a rapprochement, and the psychological transformation of attitudes which would allow it to happen, she argues, is also fundamentally necessary for the democratic future of the country.^81^

The increased appropriation of psychological explanations has been accompanied by a wider professionalization of the psy-disciplines. In 1993, historian and sociologist Nikolas Rose commented upon what he observed as the ascendancy of psychology and psychotherapy after the Velvet Revolution in Czechoslovakia. Journals, conferences and private practices were springing up across the country, signalling an expansion of these professions.^82^ Rose has argued that the psychological disciplines offer particularly useful tools for liberal and democratic societies in that they encourage individuals to self-regulate their behaviours and emotions, and has assumed that such ‘technologies of the self’ would increasingly flourish in the transition democracies of Central and Eastern Europe.^83^

Rose’s commentary underestimated the strength of these professions before the transition.^84^ Moreover, it did not sufficiently register the degree to which psychology was deployed by the socialist regime as a means to encourage self-governance, through state-run psychotherapy clinics and an array of ‘mental hygiene’ publications.^85^ Nevertheless, Rose was correct to observe that the unregulated market allowed for expansion of such practices and concepts outside of state services. The mental-health professions have also gained in prestige within the health service itself since 1989. Cyril Höschl, the psychiatrist and author of the foreword to Klára Samková’s *The Roma Question*, was able to gain support for the establishment of a National Institute for Mental Health in 2015. What Rose’s commentary did not anticipate, however, was how much psychological disciplines could come to intervene in questions of political importance, quite beyond the individual, often reflecting on collective experiences of the past and their implication for contemporary society.^86^ The place of the Roma has been no exception, even if this critique remains a minority voice. Therapeutic knowledge has been appropriated not merely as a means to regulate the individual: rather, it has offered a language through which to argue for the psychological transformation of the national community, towards the ambition of social inclusion. The motivation for this ideal has perhaps more in common with collectivist principles than the individualistic ‘advanced liberalism’ that Rose envisaged the psy-disciplines would facilitate in post-communist Europe.

## CONCLUSIONS

As the testimony from Ruben Pellar and his Dutch colleagues tells us, despite the campaigns in 1978, and then again in 1988–90, there were long silences about the problem of coercive sterilizations in the Czech lands, both before and after the fall of communism. The situation of the Romani minority more broadly has also normally been confined to a marginal position in public discussion. But when voices of dissent, and subsequently calls for therapeutic reparations, have made themselves heard, they have also sought to account for *how* such matters could have been concealed or overlooked.

What can we learn from the two discrete understandings of denial and disavowal that have emerged from these critiques? I argue that they have implications for how we might think about the emerging historiography of denial. As recent Czech history has shown, narratives that make use of a concept of the unconscious have been productive as a way to understand prejudice and injustice, and to advocate for a change in attitudes. But looking back to the communist period, we see a different way of framing the question, through the lens of phenomenology. Such an alternative, competing explication reminds us that psychoanalysis is one of a number of approaches to intervene on such questions, and that it is itself a historically situated theory of mind.

The Charter 77 movement’s texts were replete with elucidations of the processes – at a societal and individual level – which resulted in disavowals. This could be at the very basic level of noting the refusal of the state to recognize the existence of the Roma as a group within Czechoslovak society. Or, its failure to acknowledge wrongdoing through breaking the very laws that it continued to publicly uphold: from the use of contradictory and ‘alibistic’ policies, through to systematic disregard for human rights. These authors also tried to account for the more insidious and contradictory ways in which Czechoslovaks turned a blind eye to truth. They talked of how the impulse to survive and to consume could lead a whole society, *en masse*, to become seemingly mesmerized by the ritual and dogmatic phraseology of communism, in such a way as to enable this kind of injustice. These everyday acts of conformity, by necessity, involved a process of excusatory mental concealment: overlooking how the state’s actions affected one’s fellow human beings, as well as a turning away from an authentic sense of one’s own self and freedom.

Here we have a clear delineation of denial, which did not draw on psychoanalysis, or invoke unconscious defence mechanisms. Some of the concepts generated in the Czech context – most notably ‘alibism’, or the ordinary citizen who has to be ‘jarred into awareness’ about the realities they had been concealing from themselves – do, however, bear some resemblance to the psychoanalytic concept of ‘splitting’. This process, whereby a person holds two contradictory thoughts simultaneously, whilst managing to somehow keep them disconnected, is a concept which Catherine Hall and Daniel Pick argue can offer a useful addition to the historian’s toolkit when analysing texts from the past.^87^ This may well be so, but in light of the competing understandings elaborated above, one might also invite the historian interested in mental processes of denial to look further at how other worldviews may have accounted for them.^88^ The Czech case also suggests that we could still learn from looking at historical actors’ understandings of such dynamics in their own terms, within the languages of their culture and period, without assuming a pre-conceived model of mind from the outset.

Nevertheless, as this article also shows, psychoanalytic, and broader ‘therapeutic’ understandings of prejudice and disavowal have also be enthusiastically appropriated at particular historical moments. The Czech campaigns surrounding the Roma, and the coercive sterilizations in particular, are exemplary of a ‘working through’ of collective memory, trauma and denial that has become something of an imperative in Central and Eastern Europe in the years since 1989 – albeit one that has thus far failed to garner mainstream support. This has not constituted merely a wholesale importation of such discourses from other national templates. In the Czech Republic, it is also emblematic of an emerging proclivity for interweaving therapeutic concepts into socio-political debates about both past and present.

Otakar Motejl’s 2005 ombudsman’s report appears to have succeeded in bringing the practice to an end, in the Czech lands at least. That said, for many campaigners, the failure to mobilize support to pass a bill of indemnification in 2015 illustrates the ongoing refusal of the rights of the Roma as citizens of equal status, not to mention a gross minimization of the practices themselves, and a dubious excusing of societal responsibility for their continuation.^89^ Languages which explain the phenomenological or psychosocial mechanisms of denial and prejudice, and which offer imperatives for how these could be overcome, have been readily available within Czech political culture since the 1970s, yet their capacity to effect change in this arena has been limited. For all the incremental gains made over a forty-year period, the goal of a transformation of attitudes towards the Roma remains an object of struggle.

## Notes

NOTES AND REFERENCES

### Footnotes

1 Otakar Motejl, *Final Statement of the Public Defender of Rights in the Matter of Sterilisations Performed in Contravention of the Law and Proposed Remedial Measures*, Prague, 2005, p. 3.

2 Nikola Boyčev, ‘Vláda se omluvila za nezákonné sterilizace romských žen’, *Romové v České republice,* 23rd Nov. 2009 (http://romove.radio.cz/cz/clanek/22902/limit).

3 Czech Press Agency, ‘Czech Government Rejects Bill to Compensate Victims of Illegal Sterilizations’, 1 Oct. 2015 (http://www.romea.cz/en/news/czech/czech-government-rejects-bill-to-compensate-victims-of-illegal-sterilizations).

4 This article is the first to historically contextualize attempts to expose, prevent and come to terms with the coercive sterilizations, but it builds on research which has discussed the practices and their impact on the Roma community from ethnography, human-rights law, and medical ethics. See Vera Sokolova, ‘Planned Parenthood Behind the Curtain: Sterilization of Romani Women’, in Vera Sokolova, *Cultural Politics of Ethnicity: Discourses on Roma in Communist Czechoslovakia*, Stuttgart, 2008, pp. 207–40; Anna Šabatová, ‘Sterilizace romských žen prováděné v rozporu práv’, in Anna Šabatová, *Ombudsman a lidská práva*, Brno, 2008, pp. 165–74; Jiřina Stolařová, ‘Sterilizace romských žen na území bývalého Československa’, MA Dissertation, Charles University, Prague, 2010; Claude Cahn, *Human Rights, State Sovereignty and Medical Ethics: Examining Struggles around Sterilization of Romani Women*, Amsterdam, 2015; European Roma Rights Centre, *Coercive and Cruel: Sterilization and its Consequences for Romani Women in the Czech Republic, 1966–2016*, Budapest, 2016.

5 Most of the literature referenced in this article relates to Czech sources rather than Slovak, despite the fact that more sterilizations were documented within the Slovak lands than in Bohemia and Moravia. This is primarily because the reports that exposed the practices, and much of the literature which deals with the question of discrimination, were published by Czech authors (sometimes in collaboration with international NGOs). This article focuses predominantly on the question of how members of the majority, ethnically Czech, population have framed the issue. While the situation has begun to change in recent years, for much of the period under discussion the Roma community effectively had ‘subaltern’ status, with limited possibilities for representation in public discussion. For more on Slovakia, see Claude Cahn and Nidhi Trehan, ‘Time of the Skinheads: Denial and Exclusion of the Roma in Slovakia’, *East European Human Rights Review* 2: 2, 1995, pp. 153–247.

6 In addition to other contributions to this special issue, see Stanley Cohen, *States of Denial: Knowing about Atrocities and Suffering*, Cambridge, 2001.

7 Vera Sokolova, *Cultural Politics of Ethnicity*, p. 23.

8 Zoltan Barany, *The East European Gypsies: Regime Change, Marginality and Ethnopolitics*, Cambridge, 2002, p. 104.

9 Will Guy, ‘The Czech Lands and Slovakia: Another False Dawn?’, in *Between Past and Future: the Roma of Central and Eastern Europe*, ed. Will Guy, Hatfield, 2001, pp. 285–332; Zoltan Barany, *The East European Gypsies: Regime Change, Marginality and Ethnopolitics*, pp. 118–9.

10 Dena Ringold, *Roma and the Transition in Central and Eastern Europe*, Washington, 2000, p. 5.

11 European Roma Rights Centre, *Coercive and Cruel*, pp. 15–16; Cahn, *Human Rights, State Sovereignty and Medical Ethics*, pp. 44–5.

12 Cahn, *Human Rights, State Sovereignty and Medical Ethics*, p. 49.

13 Vladimír Wynnyczuk and Radim Uzel, ‘Czech Republic and Slovak Republic’, in *From Abortion to Contraception: a Resource to Public Policies and Reproductive Behavior in Central and Eastern Europe from 1917 to the Present*, ed. Henry P. David, Westport, 1999, pp. 91–120.

14 See Mary Heimann, *Czechoslovakia: the State that Failed*, Connecticut, 2011, pp. 243–77.

15 Charta 77, ‘Dokument č. 23, 14. Prosinec 1978, Dokument o postavení romských spoluobčanů předložený jako podklad k veřejné diskusi’, in *Charta 77: Dokumenty 1977–1989*, ed. Blanka Císařovská and Vilém Prečan, Prague, 2007, pp. 198–206. All translations of this document from the Czech are my own. For more on Charter 77 see Jonathan Bolton, *Worlds of Dissent: Charter 77, The Plastic People of the Universe, and Czech Dissent Under Communism*, Cambridge, MA, 2014.

16 Oxana Shevel, *Migration, Refugee Policy and State Building in Postcommunist Europe*, Cambridge, 2011, p. 196.

17 Zdeněk Pinc, *Být ovádem obce: Fragmenty ze života Zdeňka Pince*, Prague, 2010.

18 Charta 77 Dokument 23, pp. 198–99.

19 Charta 77 Dokument 23, pp. 198–99.

20 Charta 77 Dokument 23, pp. 198–99.

21 Peter Vermeersch, *The Romani Movement: Minority Politics and Ethnic Mobilization in Contemporary Central Europe*, New York, 2006, pp. 59–60.

22 OSCE, *The Helsinki Final Act*, 1 Aug. 1975 (http://www.osce.org/helsinki-final-act).

23 OSCE, *Helsinki Final Act*.

24 Charta 77 dokument 23, pp. 199–200.

25 Charta 77 dokument 23, pp. 199–200.

26 Charta 77 dokument 23, p. 201.

27 Charta 77 dokument 23, p. 204.

28 Charta 77 dokument 23, p. 204.

29 Charta 77 dokument 23, p. 205.

30 Helen O’Nions, *Minority Rights Protection in International Law: the Roma of Europe*, Abingdon, 2007, pp. 106–7.

31 Václav Havel, ‘Moc bezmocných’, Prague, 1978. Originally self-published. See Václav Havel, ‘Moc bezmocných’, in Václav Havel, *Moc bezmocných a jiné eseje*, Prague, 2012, pp. 96–223.

32 Jan Patočka, ‘What We Can and Cannot Expect from Charter 77’, in Jan Patočka, *Philosophy and Selected Writings*, ed. Erazim Kohák, Chicago, 1989, p. 343.

33 Jan Patočka, ‘What We Can and Cannot Expect from Charter 77’, p. 343.

34 Aviezer Tucker, *Philosophy and Politics of Czech Dissidence from Patočka to Havel*, Pittsburgh, 2000, pp. 2–3.

35 Martin Heidegger, *Sein und Zeit*, Halle, 1927.

36 For discussion of the phenomenological foundations of Charter 77, see chapters 6–8 in Michael Gubser, *The Far Reaches: Phenomenology, Ethics and Social Renewal in Central Europe*, Stanford, 2014; For more on Patočka, see *Jan Patočka and the Heritage of Phenomenology: Centenary Papers*, ed. Ivan Chvatík and Erika Abrams, Dordrecht, 2007.

37 Jan Patočka, *Plato and Europe*, Stanford, 2002, pp. 51–70.

38 Martin Palouš, ‘Jan Patočka’s Socratic Message for the 21st Century: Re-reading Patočka’s Charter 77 Texts Thirty Years Later’, in *Jan Patočka and the Heritage of Phenomenology*, ed. Chvatík and Abrams, p. 169.

39 Aviezer Tucker, *Philosophy and Politics of Czech Dissidence,* p. 154.

40 Václav Havel, ‘Moc bezmocných’, p. 101. The translation here is my own.

41 Václav Havel, ‘Power of the Powerless’, in Václav Havel, *Living in Truth*, London, 1987, pp. 36–122. Quotations from here onwards will be taken from this edition, translated by Paul Wilson. For more on this essay’s context and legacies, see Jiří Suk and Kristina Andělová, *Jednoho dne se v našem zelináři cosi vzbouří. Eseje o Moci bezmocných*, Prague, 2016; Paulina Bren, *The Greengrocer and his TV: the Culture of Communism after the 1968 Prague Spring*, Ithaca, 2010.

42 Václav Havel, ‘Power of the Powerless’, pp. 42–3.

43 Theodor Adorno, ‘Was bedeutet: Aufarbeitung der Vergangenheit’, in Theodor W. Adorno, *Erziehung zur Mündigkeit*, Frankfurt, 1970, pp. 10–28. See also Aleida Assmann, *Shadows of Trauma: Memory and the Politics of Postwar Identity*, New York, 2015.

44 In his 1976 essay ‘Phenomenology and Cartesianism’ (in *Jan Patočka: Philosophy and Selected Writings*, pp. 285–326), Patočka conceded that psychoanalytically oriented psychologies were a welcome ‘enrichment’ of modern science in their attempts to preserve ‘meaning’. But he rejected the Freudian unconscious:
[it] undervalues, even rejects continuity in the psychic; consciousness is in the most part *false* consciousness which closes itself off from repressed wishes of the deep psychic, passing them over in silence, transforming and deforming them. But it is not that this deformation and silencing has its own phenomenal foundation, that consciousness as such *attests* its perversion, that the turning away and flight live *in it itself –* it itself has no inkling of what is going on in it and what it means. It is as if Freud were unaware that a discontinuity between the veridical and the false presupposes a continuity, that consciousness can be false only on the basis of a capacity for truth. (p. 322) Ullman emphasizes that his ‘asubjective phenomenology does not suppose any hidden structure, any invariant foundation within human existence, on the level of the *sum*, beneath the ego,’ see Tamás Ullman, ‘Negative Platonism and the Appearance-Problem’, in *Jan Patočka and the Heritage of Phenomenology*, ed. Chvatík and Abrams, p. 77.

45 Interview with Paul Öfner, Amsterdam, 11 Jan. 2017. Öfner was aware of the topic of coercive sterilizations, from Charta 77 document 23, but says that he also heard news of it circulating at the 2nd International Congress of Roma in Geneva in April 1978.

46 Eva Davidová, ‘Medailon: Milena Hübschmannová’, *Sociologický časopis* 42: 1, 2006, pp. 203–6; Sadílková, ‘Romani teaching in the Czech Republic’, in *Roma und Travellers. Identitäten im Wandel*, ed. Erika Thurner, Elisabeth Hussl and Beate Eder-Jordan, Innsbruck, 2015, pp. 191–203; Victor A. Friedman, ‘Obituaries: Milena Hübschmannová’, *Romani Studies* 15: 2, 2005, pp. 175–9.

47 Interview with Paul Öfner.

48 Interview with Ruben Pellar; Interview with Paul Öfner.

49 Interview with Jef Helmer, Amsterdam, 17 Jan. 2017.

50 Interview with Jef Helmer; ‘Documents concerning (a study on) the sterilization of Roma women in Czechoslovakia.1988–1991’, Archive of Stichting Lau Mazirel, Folder 47, International Institute of Social History, Amsterdam.

51 Paul Öfner, Bert van der Rooij, Ruben Pellar and Zybňek Andrš, *Het Afkopen van Vruchtbaarheid*, Amsterdam, 1990; Ruben Pellar and Zybňek Andrš, ‘Report on the Examination in the issue of Sexual Sterilization of Romanies in Czechoslovakia’, unpublished document, 1990, personal papers of Ruben Pellar. The latter document constitutes the research on which the former report was based.

52 Öfner, van der Rooij, Pellar and Andrš, *Het Afkopen van Vruchtbaarheid*, pp. 32–4.

53 Indeed, the Czechoslovak government had long favoured pro-natalist policies overall, see Alena Heitlinger, *Reproduction, Medicine and the Socialist State*, Basingstoke, 1987, pp. 15–30.

54 Öfner, van der Rooij, Pellar and Andrš, *Het Afkopen van Vruchtbaarheid*, p. 35; for the original see E. Posluchová and J. Posluch, ‘Problémy planovaného rodičovstva u cigánskych spoluobčanov vo Východoslovenskom kraji’, *Zdravotnická pracovnice* 39, 1989, pp. 220–3.

55 Öfner, van der Rooij, Pellar and Andrš, *Het Afkopen van Vruchtbaarheid,* p. 37.

56 Paul Öfner, ‘Sterilisatie op zigeuners: Rapport openbaart schokkende praktijken in Tsjechoslowakije’, *Trouw*, 2 June 1990, p. 1; Paul Öfner, ‘Sterilisatiepraktijken in Tsjechoslowakije: Angst voor teveel Roma’, *o Drom*, April 1990, pp. 5–8.

57 Interview with Paul Öfner. The newspaper *Trouw* was originally founded by the Dutch Protestant resistance during the Nazi occupation.

58 Interview with Ruben Pellar.

59 Cahn, *Human Rights, State Sovereignty and Medical Ethics*, p. 34. Cahn, later an advocate for the European Centre for Roma Rights, alleges that the ‘Czech Minister of Labour and Social Affairs, Mr Zdeněk Škromach rejected the European Roma Rights Centre concerns, stating (erroneously) that “many of your statements are based on research carried out in one limited locality – Ostrava, which is a specific case to itself”’.

60 Interview with Ruben Pellar.

61 Interview with Jef Helmer.

62 Interview with Jef Helmer.

63 Interview with Ruben Pellar.

64 Ringold, *Roma and the Transition in Central and Eastern Europe*, p. 8.

65 Ringold, *Roma and the Transition in Central and Eastern Europe*, p. 8.

66 Centre for Reproductive Rights, *Body and Soul: Forced Sterilization and Other Assaults on Roma Reproductive Freedoms*, New York, 2002; Motejl, *Final Statement of the Public Defender of Rights*; Centre for Roma Rights, *Coercive and Cruel.*

67 See Cahn, *Human Rights, State Sovereignty and Medical Ethics*, p. 90, where he notes reference to the need to ‘improve the quality of the population’ in relation to Roma and family planning.

68 See Motejl, *Final Statement of the Public Defender of Rights*, pp. 68–72. Michal Šimůnek, ‘Eugenics, Social Genetics and Racial Hygiene: Plans for the Scientific Regulation of Human Heredity in the Czech Lands, 1900–1925’, in *Blood and Homeland: Eugenics and Racial Nationalism in Central and Southeastern Europe, 1900–1940*, ed. Marius Turda and Paul Weindling, Budapest, 2006, pp. 165–73; Victoria Schmidt, ‘Eugenic and Special Education in the Czech Lands in the Interwar Period: the Beginning of Segregation against Disabled and Roma’, *Social Work and Society*, 14: 1, 2006, pp. 1–19.

69 Motejl, ‘*Final Statement of the Public Defender of Rights*’, p. 3.

70 Cahn and Trehan, ‘Time of the Skinheads’, p. 155. Although this article relates to post-communist Slovakia, it traces these patterns back to the Czechoslovak state. The authors also draw on Stanley Cohen’s work on denial and atrocities.

71 David Doubek and Markéta Levínská, ‘Us and Them – what categories reveal about Roma and non-Roma in the Czech Republic’, *International Handbook of Interpretation in Educational Research*, ed. Paul Smeyers, David Bridges, Nicholas C. Burbules and Morwenna Griffiths, Dordrecht, 2015, p. 619; Dana Bittnerová, *Etnické Komunity: Romové*, Brno, 2014.

72 František Burda, *On Violence in Culture: Girardian Reflections*, Ostrava, 2013.

73 Burda, *On Violence in Culture*, p. 102.

74 See Ctibor Nečas, *Holocaust českých Romů*, Prague, 1999; Ctibor Nečas, *Romové v České republice včera a dnes,* Olomouc, 1999.

75 See also Jan Křen’s work on the implications of such politically motivated gaps: Jan Křen, *Bílá místa v naších dějinách*, Prague, 1990.

76 Markus Pape, *Nikdo vám nebude věřit*, Prague, 1997. See also Committee for the Redress of the Roma Holocaust (http://cestiromove.ecn.cz/crrheng.htm).

77 These are analyzed critically in Antony Polonsky and Joanna B. Michlic, *The Neighbours Respond: the Controversy over the Jedwabne Massacre in Poland*, Princeton, 2003; *Memory and Theory in Eastern Europe*, ed. Uilleam Blacker and Alexander Etkind, Basingstoke, 2013; *Memory and Change in Europe: Eastern Perspectives*, ed. Małgozata Pakier and Joanna Wawrzyniak, Oxford, 2015.

78 Stephen Frosh, ‘Studies in Prejudice: Theorizing anti-Semitism in the Wake of the Nazi Holocaust’, in *Psychoanalysis in the Age of Totalitarianism*, ed. Matt ffytche and Daniel Pick, Abingdon, 2016, p. 36.

79 Klára Samková, *Píšu takže žiju/žiju takže píšu,* Prague, 2013.

80 Klára Samková, *Romská otázka: psychologické příčiny sociálního vyloučení Romů*, Prague, 2012. In English translation: Klára Samková, *Roma/Gypsies from the Point of View of Existential Psychology*, Prague, 2012.

81 Samková, *Romská otázka*.

82 Nikolas Rose, ‘Experts of the Soul’, *Psychologie und Geschichte* 3: 1, 1991, p. 91.

83 Nikolas Rose, ‘Government, Authority and Expertise in Advanced Liberalism’, *Economy and Society* 22: 3, 1993, pp. 283–99; Nikolas Rose and Peter Miller, *Governing the Present*, London, 2008.

84 See Sarah Marks, ‘From Experimental Psychosis to Resolving Traumatic Pasts: Psychedelic Research in Communist Czechoslovakia, 1954–1974’, *Cahiers du monde russe* 56: 1, 2015, pp. 53–75.

85 See also Kateřina Lišková, ‘Now You See Them, Now You Don’t: Sexual Deviance and Sexological Expertise in Communist Czechoslovakia’, *History of the Human Sciences* 29, 2016, pp. 49–74.

86 Examples include work on the legacies of the trauma of political prisoners for broader society, see Kristýna Bušková and Nigel Hunt, ‘Crushing Czechoslovak Identity: Stalinist Violence and the Resistance of Czechoslovak Ex-Political Prisoners from the Communist Era’, in *Interrogation in War and Conflict: a Comparative and Interdisciplinary Analysis*, ed. Christopher Andrew and Simona Tobia, London, 2014, pp. 110–31; Klára Pinerová, ‘Forenzně-sociologická sonda do způsobu a metod vyšetřování StB v 50. letech 20. století’, *Člověk: časpois pro humanitní a společenské vědy*, 11, 2008, pp. 1–14; the essays of psychoanalytic psychotherapist and social commentator Helena Klímová in mainstream press, e.g. Klímová, ‘Agrese jako blahodární síla’, *Lidové noviny*, 7 June 2008, p. 24, and her work with the Rafael Institut, (http://www.rafaelinstitut.cz/o_nas.html).

87 See Catherine Hall and Daniel Pick, ‘Thinking about Denial’, *History Workshop Journal* 84, autumn 2017.

88 There could certainly be further elucidations of denial from non-analytic schools of psychology. One that resonates with ‘splitting’ can be found, for example, in Leon Festinger, *A Theory of Cognitive Dissonance*, Stanford, 1957.

89 Interview with Ruben Pellar. See also European Roma Rights Centre, *Coercive and Cruel*; Zbyněk Andrš and Ruben Pellar, ‘K problematice zákonnosti sterilizací romských žen v letech 1972 až 1990’, in *Proměny v postavení žen za poslední půlstoletí*, ed. Martina Horváthová, Prague, 2016.

